# Comparative Analysis of Speed-Power Performance and Sport-Specific Skills Among Elite Youth Soccer Players with Different Start Procedures

**DOI:** 10.3390/sports13100341

**Published:** 2025-10-02

**Authors:** Eduard Bezuglov, Anton Emanov, Timur Vakhidov, Elizaveta Kapralova, Georgiy Malyakin, Vyacheslav Kolesnichenko, Zbigniew Waśkiewicz, Larisa Smekalkina, Mikhail Vinogradov

**Affiliations:** 1 Department of Sports Medicine and Medical Rehabilitation, Sechenov First Moscow State Medical University, 119991 Moscow, Russia; 2 High Performance Sports Laboratory, Sechenov First Moscow State Medical University, 119991 Moscow, Russiamanuljke@gmail.com (G.M.);; 3 National Center of Sports Medicine of Federal Biomedical Agency, 115162 Moscow, Russia; 4 Institute of Sport Science, Jerzy Kukuczka Academy of Physical Education, 40-065 Katowice, Poland

**Keywords:** soccer, youth elite soccer players, sprint, change of direction

## Abstract

Accurate interpretation of physical test results is essential to objectively measure parameters both at a single point in time and throughout longitudinal assessments. This is particularly relevant for tests of speed and change of direction, which are among the most commonly used assessments for soccer players at different levels. This study aimed to quantify the impact of start-line distance (30 cm vs. 100 cm) on linear sprint splits (5–30 m), change-of-direction (COD), and T-test performance in elite youth soccer players, while also examining potential order effects. The study involved 82 youth soccer players (14–19 y; 180.68 ± 6.97 cm; 71.65 ± 7.91 kg; BMI 21.90 ± 1.57) from an elite academy, divided into two groups. The first group started trials at 30 cm from the starting line, then at 100 cm, while the second group performed in the reverse order. All participants underwent a standard sequence of tests: anthropometric measurements, 5, 10, 20, and 30 m sprints, change-of-direction running, and the T-test. The longer start (100 cm) improved sprint times with large effects tapering with distance: 5 m (Hedges’ g = 1.00, 95% CI 0.80–1.25; Δ = 0.076 s, 0.060–0.093; 6.99%), 10 m (g = 1.37, 1.14–1.68; Δ = 0.102 s, 0.086–0.119; 5.63%), 20 m (g = 1.58, 1.36–1.88; Δ = 0.112 s, 0.096–0.127; 3.66%), 30 m (g = 1.48, 1.26–1.80; Δ = 0.114 s, 0.097–0.131; 2.71%). COD also improved (rank-biserial r = 0.516, 0.294–0.717; Δ = 0.075 s, 0.034–0.116; 1.00%) and the T-test improved (g = 0.61, 0.37–0.86; Δ = 0.107 s, 0.068–0.145; 1.26%). Order effects on Δ were evident for 30 m (Welch t = −3.05, p_Holm = 0.0157, d = −0.67) and COD (MWU p_Holm = 0.0048, r = −0.43). Protocols must specify and report the start geometry; the order should be randomised or counter-balanced, particularly for 30 m and COD.

## 1. Introduction

Assessing the development of physical qualities, anthropometric indicators, and sport-specific skills plays a critical role in talent identification programmes and in evaluating the effectiveness of different training programmes for soccer players of different ages. This importance stems primarily from the need to objectively measure parameters both at a specific point in time and throughout longitudinal assessments. Instrumental methods of measurement, often considered the “gold standard”, are undoubtedly crucial for the evaluation of parameters that are essential for athletic success. Laboratory tests (e.g., isokinetic dynamometry, DXA, gas analysis treadmill) provide criterion information [1,2,3,4] but are less portable for routine academy testing. Consequently, clubs rely on validated field tests (linear sprints, COD, T-test). However, procedural heterogeneity persists (e.g., stance, gate height, and especially the distance from the start line to the first timing gate), complicating comparisons across cohorts and time. The number of such tests used to measure specific parameters can reach several dozen types [5,6,7,8].

Their main advantages are simplicity, affordability and reproducibility, making them widely applicable to soccer players of different ages. At the same time, field tests have significant drawbacks, the most important of which is the heterogeneity in implementation and reporting for even the most commonly used tests, such as sprints and vertical jumps. Another major limitation is the influence of environmental factors on test results. A notable example is the effect of wind on sprint performance. This influence is so pronounced that it is taken into account in athletics rules, which specify a wind speed limit above which an athlete’s records are not recognised [9]. Other environmental factors can also affect test results. Studies have reported similar effects from surface type [10], starting position [11], and footwear [12].

The lack of standardised protocols for the most common field tests, combined with the influence of environmental factors, makes it difficult to compare test results obtained at different times, even by the same specialists. Another problem is insufficient detail in the test descriptions within scientific studies involving soccer players. Often, such studies do not specify environmental factors such as air temperature and humidity [13,14], surface characteristics [15], or the distance to the starting line during various speed and change-of-direction tests [16,17,18].

It is clear that the distance to the start line can have a significant impact on the final result. In sprint tests, the most commonly used starting distances are 30 [14,19], 50 [20,21,22] and 100 cm [23,24]. Is it possible to compare sprint results over different distances (most commonly 5, 10, 20 and 30 m) when the starting distances are so different? It is likely that there will be differences in results, but the extent of these differences remains unknown. To the best of the authors’ knowledge, no studies have specifically investigated the effect of start distance on sprint and change of direction tests in elite soccer players. We were only able to identify two studies that investigated the effect of different starting distances on sprinting, change of direction and dribbling performance in adult university athletes [25] and youth soccer players [26]. This practical gap motivates the present work, as quantifying the effect can help practitioners interpret and harmonise results obtained under different protocols. We selected 30 cm and 100 cm because both are widely used in academy testing and bracket the practical range—from a near-standing, toe-on-line stance to a short rolling approach—allowing us to isolate the effect within current practice.

The cohort comprised elite academy youth (U15–U19) tested under routine club procedures, providing estimates directly applicable to high-performance development programmes.

Purpose. We quantified the effect of start-line distance (30 cm vs. 100 cm) on 5–30 m splits, COD, and T-test in elite youth players, and tested for order effects.

Hypotheses. (i) The 100 cm start would yield faster times than 30 cm, with larger effects at shorter splits (5–10 m) due to greater approach velocity and shorter early ground contacts, and smaller effects by 30 m; (ii) effects on COD and the T-test would be smaller than for linear sprints; (iii) order effects would be negligible under 3 min recoveries.

## 2. Materials and Methods

### 2.1. Ethical Approval

This study was conducted in accordance with the Declaration of Helsinki. All participants and their legal guardians signed voluntary informed consent forms and were informed about the potential risks associated with participation. The study protocol was approved by the official Local Ethics Committee of Sechenov First Moscow State University (protocol number 06–21, 7 April 2021).

### 2.2. Participants and Allocation

The study involved 82 youth soccer players from an elite soccer academy, aged 14–19 years (mean age: 16.65 ± 1.33 years, height: 180.68 ± 6.97 cm, weight: 71.65 ± 7.91 kg, BMI: 21.90 ± 1.57 kg/m^2^). Players were assigned 1:1 to start order (S30→S100 vs. S100→S30) using random allocation. Group sizes: 41 vs. 41 (T-test: 40 vs. 41 due to one incomplete case). The first group (n = 41) performed the initial attempt with a start distance of 30 cm from the starting line (S30), followed by a second attempt at 100 cm (S100). The second group (n = 41) performed the reverse order, starting initially at 100 cm and then at 30 cm. The groups were comparable in chronological age and training experience to ensure consistency.

### 2.3. Inclusion Criteria

- Participation in regular soccer training for at least 12 months;
- Consistent membership of high-level organised soccer teams.

### 2.4. Exclusion Criteria

- Presence of injury or illness resulting in absence from more than three training sessions within three months prior to the study;
- Withdrawal from the study at any time;
- Persistence of an injury that prevented full participation in the study.

### 2.5. Testing Conditions

All tests were conducted between 10:00 and 15:00 under controlled conditions (temperature not exceeding 23 °C, humidity below 60%) on an artificial soccer surface familiar to the athletes. Participants wore their regular training attire, including shorts, shirts, and soccer boots. All participants had prior experience with the tests included in the study. No intense training sessions were performed 48 h before the testing day, with participants having either two rest days or one rest day followed by a light training day. On the testing day, participants were briefed on the rules and procedures of the tests. Before performing the first sprint, all players completed a familiar warm-up routine under the guidance of their coaches, which lasted 12–15 min. To minimise the risk of acute muscle fatigue, at least 3 min elapsed between the end of the warm-up and the start of the high-intensity test protocol. The testing sequence was consistent and proceeded as follows: measurement of anthropometric parameters (height and weight); a 30-m sprint with splits recorded at 5, 10, and 20 m; COD running; and a T-test. All tests were conducted by the same testing team using the same equipment to ensure consistency.

### 2.6. Anthropometric Measurements

The height of the participants was measured using a portable stadiometer (Seca-217, Seca, Hamburg, Germany) positioned on a solid, flat surface. Height was measured by a specialist with specific training in these methods, in strict accordance with the guidelines of the International Society for the Advancement of Kinanthropometry. Body weight was measured using a standing scale (Seca-813, Seca, Hamburg, Germany). Participants were weighed wearing shorts and a T-shirt, without shoes, at the same time of day for all measurements.

### 2.7. Testing Battery

**Figure 1:**
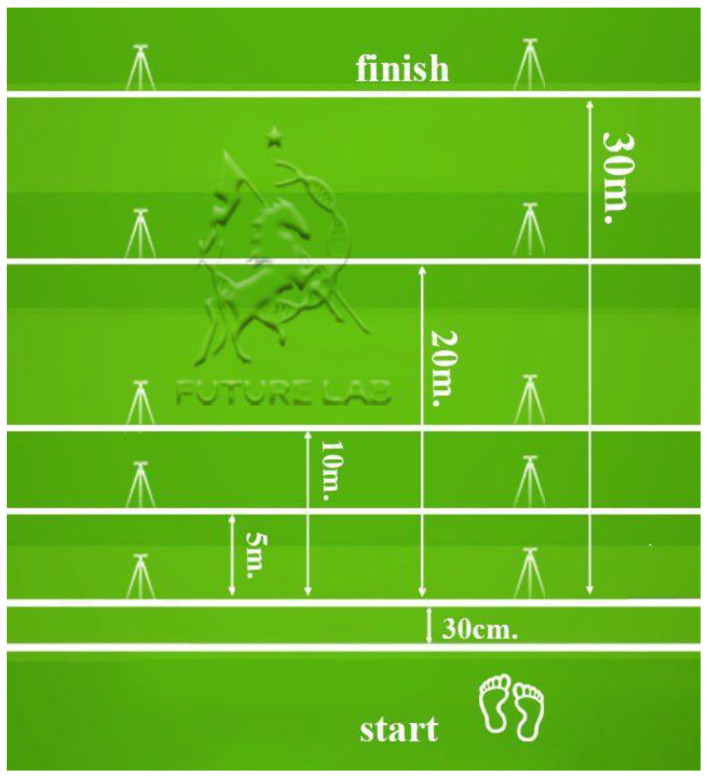
Sprint 30 m with split 5, 10, 20 m.

**Figure 2:**
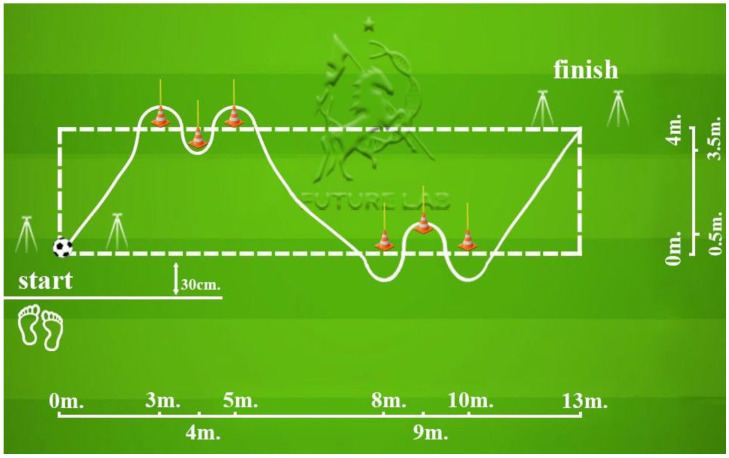
Change of direction running.

**Figure 3:**
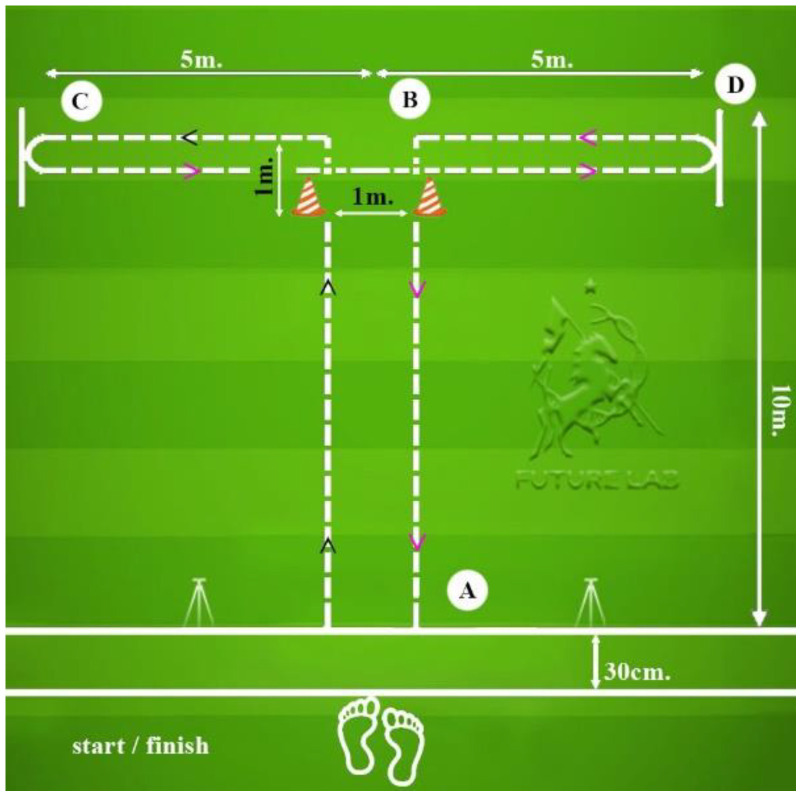
T-test.

The testing protocol included a 30-m sprint with split times at 5, 10, and 20 m (Figure 1). The COD course and T-test layouts are shown in Figure 2 and Figure 3; total path lengths were ≈27 m (COD) and ≈40 m (T-test), with first decelerations at ≈4 m and ≈10 m, respectively; timing gates were set at ~100 cm.

### 2.8. Participant Instructions and Equipment

Participants were instructed to perform all tests with maximum effort. No verbal encouragement was given by the research coordinators during the tests. The SmartSpeed Plus timing system (VALD Performance, Newstead, QLD, Australia) was used to measure the results of the sprint, COD and T-test. Timing gates were placed at a height of 100 cm above the surface. This system minimises timing errors by eliminating premature starts caused by unintentional hand movements by participants. Participants performed the sprint, COD and T-test twice, with a 3-min recovery period between trials to reduce the risk of acute muscle fatigue. This is a standard practice to minimise fatigue. The start stance was a two-point split stance; the lead foot was on the start line, the rear foot approximately shoulder-width behind; arms free; self-initiated start; gates at ~100 cm height. Start distance was defined as the horizontal distance from the lead foot’s toe to the plane of the first gate: 30 cm (S30) or 100 cm (S100) [11].

### 2.9. Statistical Analysis

All statistical analyses were conducted using Python (version 3.11.9). The primary packages used included Pandas (version 2.3.2) for data manipulation, SciPy (version 1.15.1) for statistical testing, Statsmodels (version 0.14.2) for advanced statistical modelling, Seaborn (version 0.13.2) and Matplotlib (version 3.8.4) for visualisations, and Pingouin (version 0.5.5) for effect size calculations.

For each performance metric, paired comparisons were made between the two start procedures (S30 vs. S100). The normality of each performance metric was assessed using the Shapiro–Wilk test (two-sided, α = 0.05). If the paired differences were approximately normal, we used paired *t*-tests; otherwise, we used the Wilcoxon signed-rank test. Effect sizes for paired *t*-tests were reported as Hedges’ *g* (small-sample corrected Cohen’s *d*), interpreted using Cohen’s conventions (0.20 small, 0.50 medium, 0.80 large). For Wilcoxon tests, effect sizes were reported as the rank-biserial correlation (*r*_rb_), interpreted using commonly adopted thresholds (≈0.10 small, ≈0.30 medium, ≈0.50 large). For all paired outcomes, we also report the mean delta (Δ = S30–S100) with its 95% confidence interval, and the per cent change (Δ% relative to S30). 95% CIs for effect sizes were obtained via non-central *t* (Hedges’ *g*) or bootstrap resampling (rank-biserial; 5000 paired resamples).

To assess whether the order of conditions influenced results, we conducted two complementary analyses:

(i) Between-group comparisons within each condition (S30 and S100 separately) using Welch’s independent *t*-test (if both groups were approximately normal) or the Mann–Whitney U test (otherwise), with effect sizes as Cohen’s *d* (independent) or rank-biserial correlation, respectively;
(ii) Between-group comparisons of the within-subject delta (Δ = S30–S100), which we treat as the primary order-effect test because it directly tests whether the S30→S100 change depends on the order group.

Given multiple outcomes, we controlled family-wise error using the Holm–Bonferroni procedure separately within each family (paired comparisons; order-effect analyses). Alongside *p*-values, we report minimal detectable effects (MDE) for paired outcomes (α = 0.05, 1 − β = 0.80), both in standardised units (Cohen’s *d*_paired_) and converted to seconds using the SD of paired differences. For paired *t*-tests, we additionally report achieved power; post hoc power is not reported for Wilcoxon tests.

Descriptive statistics for all performance metrics were calculated, including mean ± standard deviation (SD) and median with interquartile range (IQR) for each start procedure. This dual representation allows for a clear understanding of both central tendency and distribution spread. The level of significance for all tests was set at α = 0.05. Visual representations included: box plots to compare distributions between start conditions (30 cm vs. 100 cm), paired difference plots to illustrate individual differences, with lines connecting the group means.

We additionally examined individual differences by correlating baseline performance (S30) with the within-subject delta (Δ = S30–S100) using Pearson and Spearman coefficients with 95% CIs (5000 bootstrap resamples).

One athlete was unable to complete the T-test measurements under both start conditions due to an injury. Therefore, this athlete was excluded from the analysis involving the T-test to maintain data integrity and to avoid introducing bias.

## 3. Results

Table 1 presents the descriptive statistics for each performance metric under the two starting procedures. The results are reported as mean ± SD and median [IQR] (Table 1). Within-subject analyses used n = 82 for sprint splits and COD, and n = 81 for the T-test (one incomplete case).

**Figure 4:**
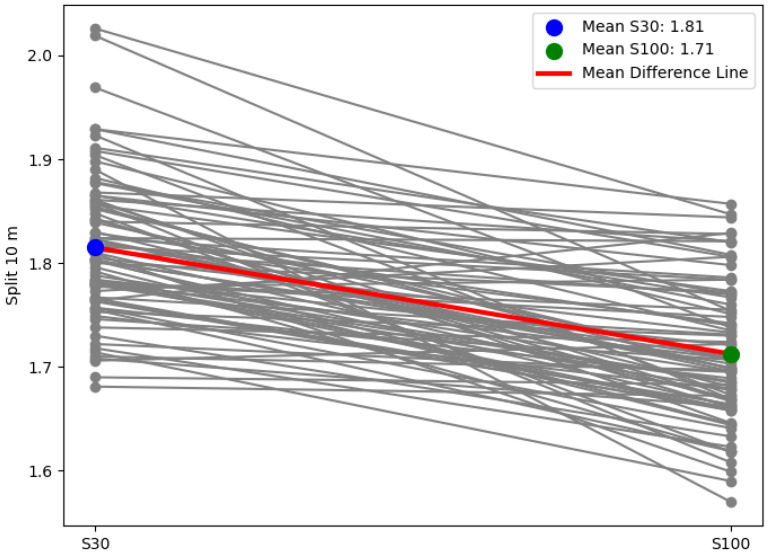
Paired difference plot for split 10 m.

**Figure 5:**
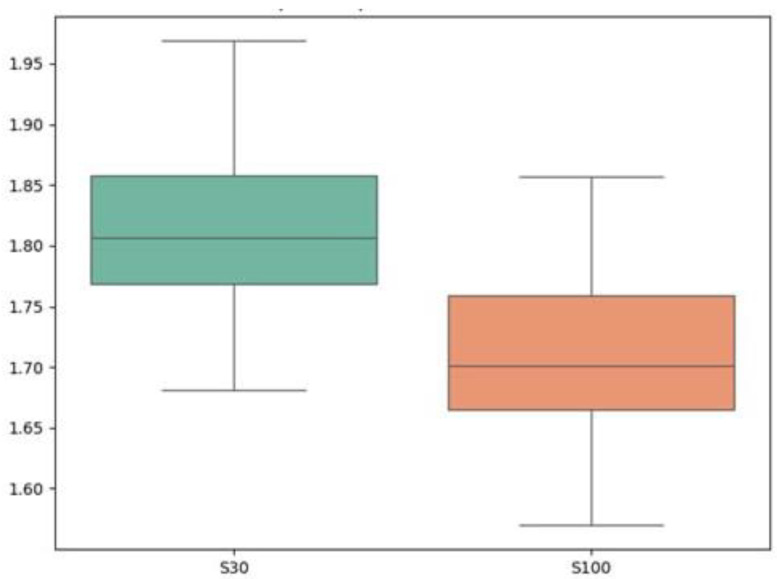
Box plot of Split 10 m: S30 vs. S100.

Paired difference plots (Figure 4) and box plots (Figure 5) illustrate the differences in performance metrics between the two starting procedures. The paired difference plots clearly show the individual changes between S30 and S100 for each athlete, with the mean difference line highlighting the overall trend. The box plots also confirm the differences in distribution between the two starting conditions.

Table 2 shows the results of the paired comparisons between the two starting procedures. Each metric is accompanied by the raw *p*-value and the Holm-Bonferroni adjusted *p*-value (Table 2).

### 3.1. Effect Sizes and Power Analysis

The largest effects were observed for the sprint splits, with large Hedges’ *g* across all distances (5 m: 1.00; 10 m: 1.37; 20 m: 1.58; 30 m: 1.48). COD showed a large effect (rank-biserial *r* = 0.52), and the T-test a medium effect (*g* = 0.61). For paired *t* outcomes, achieved power was ~1.00 for all metrics; power is not reported for Wilcoxon tests. 95% CIs for effect sizes (and for mean deltas) are provided in the Supplementary table (Table S1). Representative 95% CIs in the main outcomes were: 10 m (Hedges’ *g* = 1.37, 1.14–1.68), 30 m (Hedges’ *g* = 1.48, 1.26–1.80), and COD (rank-biserial *r* = 0.52, 0.29–0.72).

### 3.2. Magnitude of the S100 Effect on Sprint Performance

The 100 cm start procedure had a significant positive effect on sprint performance, particularly over shorter distances. The percentage improvement in sprint times between S30 and S100 was greatest for the 5 m sprint (6.99%). As the sprint distance increased, the magnitude of the improvement decreased: 5.63% for the 10 m sprint, 3.66% for the 20 m sprint and 2.71% for the 30 m sprint.

Baseline performance (S30) correlated positively with Δ at 5–10 m (e.g., 5 m Pearson *r* = 0.70, 95% CI 0.57–0.80), indicating that slower athletes gained more in absolute seconds from the longer start distance.

### 3.3. Order Effects Analysis

We evaluated possible order effects in two complementary ways:

(1) Condition-wise comparisons (S30 and S100 analysed separately)—summarised in Table 3; and
(2) Within-subject delta comparisons (Δ = S30–S100 between order groups)—our primary order-effect test, detailed in the paragraph below and in Supplementary Table S2.

For the condition-wise tests (Table 3), after adjustment for multiple comparisons, no significant order effects were found for any metric (all Holm-adjusted *p* ≥ 0.617). Although some raw *p*-values (e.g., 30 m sprint in the S30 condition, *p* = 0.051) suggested potential differences, these were not significant after Holm–Bonferroni correction.

For the within-subject change (Δ = S30–S100) between order groups (primary test), significant order effects emerged after Holm adjustment for 30 m (Welch *t* = −3.05, *p* = 0.0031, *p*_Holm_ = 0.0157, Cohen’s *d* = −0.67; mean Δ_30-first_ = 0.0895 s, mean Δ_100-first_ = 0.1384 s) and for COD (MWU *p* = 0.00080, *p*_Holm_ = 0.0048, *r* = −0.43; mean Δ_30-first_ = 0.1358 s, mean Δ_100-first_ = 0.0141 s). No Δ-order differences were observed for 5–20 m sprints or the T-test (all adjusted *p* ≥ 0.21). Full Δ-based results are provided in Supplementary Table S2.

## 4. Discussion

The results indicate that varying the distance to the start line in speed tests significantly influences the results. The magnitude of this influence varied across the different segments, peaking at 6.99% improvement in the 5-m sprint and decreasing to 2.71% at 30 m. A smaller, but still meaningful effect was also present for COD (Δ ≈ 0.075 s; rank-biserial *r* ≈ 0.52), whereas the T-test showed a medium effect (Δ ≈ 0.107 s; Hedges’ *g* ≈ 0.61). Although this direction of effect is intuitive, the magnitude is operationally important: even a ~7% shift at very short splits can reorder athletes near selection thresholds and bias longitudinal interpretation when protocols differ across testing days or teams. Accordingly, our estimates provide concrete bounds to harmonise or contextualise legacy datasets when the start-line distance has not been held constant.

The tests used in this study are highly relevant to both youth and adult soccer [8,14,27] and are widely used in talent identification programmes, pre-contract evaluations [28,29,30,31], training sessions, rehabilitation processes and in decision-making regarding return to play after injury [32,33,34,35]. Dynamic assessment of sport-specific skills plays a crucial role as a key parameter in monitoring and evaluating progress [31,36] due to their importance for athletic success [37,38,39]. In practice, small procedural differences (e.g., stance, gate height and, as shown here, distance from the start line to the first timing gate) can meaningfully alter outcomes; thus, strict reporting and standardisation of these details remain essential.

As mentioned earlier, only two studies have investigated the impact of starting line distance on sprint performance outcomes [25,26]. In the first study, 13 male sport students aged 25.6 ± 1.8 years participated in a 5-m sprint, positioning their front foot at distances of 30, 50, and 100 cm from the starting line. The results showed that sprint times were significantly faster for the 1.0 m starting distance (0.98 ± 0.06 s) compared to the 0.5 m (1.05 ± 0.07 s) and 0.3 m (1.09 ± 0.08 s) starting distances (*p* < 0.001). The authors attributed these findings to the observation that athletes were already accelerating before crossing the initial timing gate [25]. In the study by Gioldasis et al., involving 12 amateur soccer players aged 11.41 ± 0.52 years, the authors also found that 15-m sprint times tended to decrease from 3.28 s with a standing start position (30 cm) to 2.82 s with a flying start (3 m) [26]. However, no studies to date have investigated the effect of different starting line distances on sprint performance over 10 m or more in professional soccer players under highly ecological conditions (wearing soccer boots on artificial turf). In particular, sprints ranging from 15 to 20 m are the most common in competitive matches [40,41]. Considering that sprint tests are widely used in the practice of various sports organisations, it is important to note that the lack of strict adherence to test methods and their standardisation can significantly affect the subsequent interpretation of results as well as their comparability [36]. The present findings extend this literature by quantifying how much performance shifts across commonly used splits (5–30 m) under ecologically valid conditions, providing effect sizes and confidence intervals that can be used to benchmark or adjust field-testing practices.

It is important to note that the difference in results is most pronounced at the 5 m mark (6.99%) and least significant at the 30 m mark (2.71%). These results are not surprising, as the athlete’s speed at the start line is higher at S100 than at S30, making this factor crucial at shorter distances. Practitioners should take these findings into account when evaluating the results of sprint tests conducted at different starting distances. At the same time, the start distance had a large effect on COD (rank-biserial *r* ≈ 0.52; Δ ≈ 0.075 s) and a medium effect on the T-test (Hedges’ *g* ≈ 0.61; Δ ≈ 0.107 s). Consistent with this pattern, relative (%) effects shrink with distance, whereas absolute time savings remain meaningful at short splits; together, these patterns underscore the need to fix and report start-line distance in any longitudinal or between-group comparisons. At the same time, the start distance had a large effect on COD (rank-biserial *r* ≈ 0.52; Δ ≈ 0.075 s) and a medium effect on the T-test (Hedges’ *g* ≈ 0.61; Δ ≈ 0.107 s). This could be due to both the distances covered in these tests (approximately 40 m in the T-test and 27 m in the COD) and the essential role of coordination in successful execution. It is also important to note that any potential advantage offered by the S100 start is nullified after the first deceleration, which occurs at approximately 10 m in the T-test and 4 m in the COD.

A longer pre-gate distance plausibly increases approach velocity at the first timing plane and shortens early ground contact times, improving horizontal impulse during the initial acceleration steps. Benefits shrink with distance as velocities converge and the contribution of early acceleration diminishes. For COD and T-test, start-distance effects likely depend on entry speed versus braking demand and coordination quality; the first deceleration (≈10 m in the T-test; ≈4 m in COD) can attenuate gains from higher approach speed, consistent with our smaller net effects compared with linear splits.

In the previously mentioned study by Gioldasis et al., an improvement in the average COD time was observed with a flying start (3 m) compared to a standing start (30 cm), with times of 4.32 and 4.52 s, respectively [26]. The observed results can be explained by the significant difference in the starting distances compared: 270 cm versus 70 cm in our study, which allows for a higher speed when crossing the starting line. Additionally, these differences may stem from variations in the participants’ movement patterns during the tests. Our data also showed positive baseline–Δ associations at short splits (e.g., 5 m Pearson *r* ≈ 0.70, 95% CI 0.57–0.80), suggesting that slower athletes benefited more in absolute seconds from a longer start; this aligns with the dominant role of early-acceleration mechanics and may help practitioners individualise interpretation of short-sprint testing.

When comparing the within-subject change (Δ = S30–S100) between order groups, significant order effects were observed for the 30 m split (Welch *t* = −3.05, *p* = 0.0031, *p*_Holm_ = 0.0157, Cohen’s *d* = −0.67; mean Δ_30-first_ ≈ 0.0895 s, mean Δ_100-first_ ≈ 0.1384 s) and for COD (Mann–Whitney U *p* = 0.00080, *p*_Holm_ = 0.0048, *r* = −0.43; mean Δ_30-first_ ≈ 0.1358 s, mean Δ_100-first_ ≈ 0.0141 s). This indicates that learning/familiarisation, arousal, or subtle fatigue/coordination interactions can influence protocol transitions even with ≥3 min passive recoveries; future work should incorporate within-athlete counter-balancing and physiological markers (e.g., HR, RPE) to verify recovery sufficiency. Accordingly, we recommend randomised or counter-balanced order whenever feasible, and clear reporting of the order used when it is not.

### 4.1. Practical Recommendations

For linear sprints and COD/T-test in academy settings: (i) Define the start-line distance as toe-to-gate-plane and fix it (e.g., 30 cm or 100 cm); (ii) Report start distance, gate height (~100 cm), stance, order, trial count, and environment (surface, temp, humidity); (iii) Randomise/counter-balance order within sessions; (iv) Use ≥3 min passive recoveries and consider RPE/HR logging; (v) When comparing to legacy data with different start distances, interpret differences using the magnitude estimates provided here (e.g., ≈7% at 5 m tapering to ≈3% at 30 m).

### 4.2. Limitations and Directions for Future Research

A limitation of this study is its reduced ecological validity, as most sprints in soccer are not linear but curvilinear [23]. This also applies to COD performance, which in soccer is often associated with dribbling. Further limitations include the absence of biological maturation indices (e.g., PHV offset; analyses adjusted only for chronological age) and the lack of a dedicated test–retest reliability assessment within this cohort. Psychophysiological factors (e.g., motivation, arousal, perceived exertion) and technique variability were not measured, although instructions were standardised and no verbal encouragement was given. Start-order was randomised at the group level within a single session; residual learning/familiarisation or fatigue effects cannot be fully excluded—consistent with the Δ-based order findings in the Results. One athlete did not complete the T-test; thus, T-test analyses used n = 81, while all other outcomes used n = 82. Positional role and training load were not recorded and may contribute to between-athlete variability. Finally, the single-academy context may limit the generalisability of absolute times, although the relative influence of protocol (start-line distance) is likely to transfer.

Future research should investigate the influence of other environmental factors on soccer-related tests, including humidity and temperature, surface characteristics, and soccer footwear (e.g., cleat type and material). In addition, future work should formalise start-geometry reporting, randomise/counter-balance order (especially for 30 m and COD), include maturation profiling and independent reliability studies, and test whether simple correction factors can harmonise datasets collected under different start-line distances.

## 5. Conclusions

The starting distance has a large and practically meaningful impact on linear sprints, most pronounced at short splits (≈7% at 5 m; 5.6% at 10 m; 3.7% at 20 m; 2.7% at 30 m). The start procedure also had smaller but significant effects on COD (Δ ≈ 0.075 s; r_rb ≈ 0.52) and the T-test (Δ ≈ 0.107 s; g ≈ 0.61). We found order effects on the delta for 30 m (p_Holm = 0.0157) and COD (p_Holm = 0.0048), indicating that sequence can influence the observed magnitude. Practitioners should fix and report start-line distance, randomise/counter-balance order (particularly for 30 m and COD), and document gate height, stance, and environment to support comparison and longitudinal tracking.

## Figures and Tables

**Table 1:** Descriptive Statistics of Performance Metrics for Two Start Procedures (S30 and S100).

Metric	S30: Mean ± SD	S30: Median [IQR]	S100: Mean ± SD	S100: Median [IQR]
Sprint 30 m (s)	4.21 ± 0.14	4.19 [4.16, 4.35]	4.10 ± 0.14	4.07 [4.03, 4.27]
Sprint 20 m (s)	3.05 ± 0.10	3.04 [2.96, 3.16]	2.94 ± 0.10	2.92 [2.90, 3.03]
Sprint 10 m (s)	1.81 ± 0.07	1.81 [1.73, 1.90]	1.71 ± 0.07	1.70 [1.66, 1.82]
Sprint 5 m (s)	1.09 ± 0.07	1.09 [1.02, 1.15]	1.02 ± 0.06	1.02 [0.98, 1.10]
COD (s)	7.49 ± 0.31	7.47 [7.32, 7.88]	7.42 ± 0.35	7.38 [7.20, 7.78]
T-test (s)	8.48 ± 0.31	8.50 [8.30, 8.74]	8.38 ± 0.30	8.35 [8.20, 8.60]

**Table 2:** Paired comparison of performance metrics between two start procedures (S30 vs. S100).

Metric	Test Type	Test Statistic	*p*-Value	Corrected *p*-Value (Holm)	Effect Size	Power
Sprint 30 m (s)	Paired *t*-test	13.52	1.78 × 10^−22^	8.92 × 10^−22^	1.48	1.00
Sprint 20 m (s)	Paired *t*-test	14.47	3.61 × 10^−24^	2.17 × 10^−23^	1.58	1.00
Sprint 10 m (s)	Paired *t*-test	12.52	1.23 × 10^−20^	4.93 × 10^−20^	1.37	1.00
Sprint 5 m (s)	Paired *t*-test	9.16	3.76 × 10^−14^	1.13 × 10^−13^	1.00	1.00
COD (s)	Wilcoxon Signed-Rank Test	824	5.03 × 10^−5^	5.03 × 10^−5^	0.52	N/A
T-test (s)	Paired *t*-test	5.51	4.31 × 10^−7^	8.62 × 10^−7^	0.61	1.00

Effect sizes: Hedges’ *g* for paired *t*-tests; rank-biserial correlation (*r*_rb_) for Wilcoxon.

**Table 3:** Condition-wise order comparisons (S30 and S100 analysed separately).

Meric	Test Type	Test Statistic	*p*-Value	Corrected *p*-Value (Holm)	Mean (30 cm First)	Mean (100 cm First)	Effect Size (Order Effect)
Sprint 30 m (S30)	Mann–Whitney U test	630	0.051	0.617	4.18 ± 0.13	4.24 ± 0.14	0.25
Sprint 30 m (S100)	Mann–Whitney U test	837	0.978	1	4.09 ± 0.15	4.10 ± 0.14	0.00
Sprint 20 m (S30)	Mann–Whitney U test	683.5	0.147	1	3.03 ± 0.09	3.07 ± 0.10	0.19
Sprint 20 m (S100)	Mann–Whitney U test	834	0.956	1	2.94 ± 0.10	2.94 ± 0.10	0.01
Split 5 m (S30)	Independent *t*-test	−0.52	0.604	1	1.09 ± 0.06	1.10 ± 0.08	−0.12
Split 5 m (S100)	Mann–Whitney U test	894.5	0.620	1	1.02 ± 0.05	1.01 ± 0.06	−0.06
Split 10 m (S30)	Independent *t*-test	−1.38	0.170	1	1.80 ± 0.06	1.83 ± 0.07	−0.31
Split 10 m (S100)	Independent *t*-test	−0.25	0.799	1	1.71 ± 0.06	1.71 ± 0.07	−0.06
COD (S30)	Independent *t*-test	0.70	0.489	1	7.51 ± 0.28	7.47 ± 0.34	0.15
COD (S100)	Mann–Whitney U test	787	0.623	1	7.38 ± 0.28	7.45 ± 0.40	0.06
T-test (S30)	Independent *t*-test	−0.35	0.727	1	8.47 ± 0.28	8.49 ± 0.36	−0.08
T-test (S100)	Independent *t*-test	−0.18	0.861	1	8.37 ± 0.31	8.38 ± 0.30	−0.04

Effect sizes are computed as (30-first minus 100-first); positive values indicate higher (slower) times in the 30-first group.

## Data Availability

The datasets used and/or analysed during the current study are available from the corresponding author on reasonable request.

## Supplementary Materials

The following supporting information can be downloaded at: https://www.mdpi.com/article/10.3390/sports13100341/s1, Table S1: Within-subject comparisons (S30 vs. S100) with effect sizes and confidence intervals; Table S2: Order effects on the within-subject delta (Δ = S30–S100) by metric (primary order-effect analysis).
