# Supplementary material for: Comparative Analysis of Speed-Power Performance and Sport-Specific Skills Among Elite Youth Soccer Players with Different Start Procedures
Source: Sports (Basel). 2025 Oct 2;13(10):341. doi: 10.3390/sports13100341 (PMC12568230; doi:10.3390/sports13100341)
Supplement: Supplementary file 1 [file sports-13-00341-s001.zip › sports-3845262-supplementary.pdf]

Supplementary Table S1. Within-subject comparisons (S30 vs S100) with effect sizes and confidence intervals.

Metric	n	Test	Effect size type	Effect size	ES 95% CI (low–high)	Δ (S30–S100), s	Δ 95% CI (s)	Δ %, rel. to S30	Δ % 95% CI	p-value	Corrected p (Holm)	Achieved power	MDE (d)	MDE (s)
Split 5 m	82	Paired t-test	Hedges’ g (paired)	1.00	0.80–1.25	0.076	0.060–0.093	6.99	5.47–8.50	3.76×10^{-14}	1.13×10^{-13}	1.000	0.313	0.024
Split 10 m	82	Paired t-test	Hedges’ g (paired)	1.37	1.14–1.68	0.102	0.086–0.119	5.63	4.74–6.53	1.23×10^{-20}	4.93×10^{-20}	1.000	0.313	0.023
Sprint 20 m	82	Paired t-test	Hedges’ g (paired)	1.58	1.36–1.88	0.112	0.096–0.127	3.66	3.16–4.17	3.61×10^{-24}	2.17×10^{-23}	1.000	0.313	0.022
Sprint 30 m	82	Paired t-test	Hedges’ g (paired)	1.48	1.26–1.80	0.114	0.097–0.131	2.71	2.31–3.10	1.78×10^{-22}	8.92×10^{-22}	1.000	0.313	0.024
COD	82	Wilcoxon Signed-Rank	Rank-biserial r	0.52	0.29–0.72	0.075	0.034–0.116	1.00	0.45–1.55	4.98×10^{-5}	4.98×10^{-5}	—	0.313	0.059
T-test	81	Paired t-test	Hedges’ g (paired)	0.61	0.37–0.87	0.107	0.068–0.145	1.26	0.80–1.72	4.31×10^{-7}	8.62×10^{-7}	1.000	0.315	0.055

Effect sizes: Hedges’ g for paired t-tests; rank-biserial r for Wilcoxon. 95% CIs for ES from non-central-t (g) or bootstrap (r). 95% CIs for Δ and Δ% from paired t intervals. Achieved power reported only for paired t-tests. MDE for paired outcomes (α = 0.05, 1−β = 0.80).

Supplementary Table S2. Order effects on the within-subject delta (Δ = S30–S100) by metric (primary order-effect analysis).

Metric	Test	Test statistic	p-value	Corrected p (Holm)	Effect size (type)	Mean Δ (30-first), s	Mean Δ (100-first), s
Split 5 m	Independent t-test (Welch)	t = −0.677	0.500	1.000	d = −0.150	0.0706	0.0820
Split 10 m	Independent t-test (Welch)	t = −1.052	0.296	0.888	d = −0.232	0.0937	0.1109
Sprint 20 m	Independent t-test (Welch)	t = −1.968	0.0525	0.210	d = −0.435	0.0968	0.1267
Sprint 30 m	Independent t-test (Welch)	t = −3.047	0.00313	0.0157	d = −0.673	0.0895	0.1384
COD	Mann–Whitney U	U = 1202.5	0.000800	0.00480	r = −0.431	0.1358	0.0141
T-test	Independent t-test (Welch)	t = −0.330	0.742	1.000	d = −0.073	0.1003	0.1132
